# Heterogeneity of crisis communication practices in hotels: Anti-COVID-19 measures on Adriatic beach hotels’ websites

**DOI:** 10.1177/13567667231164454

**Published:** 2023-03-27

**Authors:** Metod Šuligoj

**Affiliations:** University of Primorska, Slovenia

**Keywords:** Beach hotels, Adriatic, COVID-19 measures, health crises, crisis communication

## Abstract

Based on situational crisis communication theory, this study designs a research framework to identify differences in crisis communication practises on beach hotel websites in different Adriatic countries and between three consecutive summer seasons due to the COVID-19 pandemic. A qualitative inductive content analysis of official pandemic-related guidelines/recommendations from national and international competent organizations was carried out. Subsequently, an overlooked repeated measures design with deductive quantitative content analysis of crisis communications on hotel websites during the three summer seasons was conducted. Employing the McNemar test, the Cochran's Q-test and Friedman test with post-hoc comparisons, it was discovered that (1) beside the bolstering strategy, the new ‘ignore strategy’ of crisis communication was also identified, which has many implications, (2) communication of anti-COVID-19 measures is statistically significant and relatively strongly associated with the country in which hotels are located, and (3) the number of different announcements on anti-COVID-19 measures is significantly different only between two seasons.

## Introduction

The impact of COVID-19 on national economies including tourism is unprecedented (Hidalgo et al., 2022; Škare et al., 2021). Crisis thus negatively affect hotels’ occupancy (Hidalgo et al., 2022; Rittichainuwat and Chakraborty, 2009), marketing and sales (Guo et al., 2022; Jiang and Wen, 2020), communication (Kwok et al., 2021; Wong et al., 2021), employees (Aguiar-Quintana et al., 2021; Šuligoj, 2022) and other segments of a business. All this calls researchers to investigate the crisis management of hotels in tourism (Aldao et al., 2022; Le and Phi, 2021). Accordingly, Hsieh et al. (2021) point to the need for research on revised marketing and promotion strategies of hotels in response to the pandemic; Atasoy et al. (2022), who focused on hotel chains and their COVID-19-related announcements disseminated via websites, suggest more research on the different stages of the pandemic, as well as increasing the number and scope of hotels. Wong et al. (2021) also direct to more research on crisis and COVID-19 communication, which makes the present research relevant and topical. Hence, Le and Phi (2021), Zizka et al. (2021) and Kim et al. (2023) also suggest focusing on non-English (international) references/sources/cases and comparative studies to become familiar with practices in the wider international setting that depend on different crisis situations. They also suggest research into different types of hotels. Accordingly, there is a significant lack of knowledge about crisis communication via hotel websites during a long-lasting (global) health crisis. In this context, we also know little about how different types of hotels behaved in various destinations. For example, there are about 3.5 independent hotels for every chain property in Europe (Ledsham, 2022), but no good research on their website crisis communication during the COVID-19 pandemic was found. Researchers should provide relevant analysis to stakeholders such as owners, managers, marketers and others, and place the findings in an appropriate theoretical framework to better understand and evaluate them and be better prepared for future (global) crises.

Filep et al. (2022) point to interdisciplinary research with more data over longer periods of time. Additionaly, Kuščer et al. (2021) recommend comparison of destinations to provide further insight into the management of the different waves of pandemic. Accordingly, this paper thus focuses on the six Adriatic countries as one of the typical coastal tourist areas of the Mediterranean (Cori, 1999); they are among the most tourism-dependent countries with adequate coastal accommodation in Europe and are therefore vulnerable to health shocks (*Eurostat*, 2021*b*; MacDonald et al., 2020). Scholars such as Butler (2022), Payne et al. (2022) and Mancinelli et al. (2022) also consider the Adriatic countries an appropriate tourism area to study the current crisis. Beach hotels, so typical of coastal areas, have been overlooked so far, although beach tourism has received attention in research, see Zielinski and Botero (2020).

In summary, the present research builds on previous studies and focuses on the COVID-19-related communication practices detected during the summer seasons among Adriatic beach hotels, which are directly/highly dependent on the summer season. More specifically, the main purpose is to identify differences (1) in crisis communication practises on the website of beach hotels in different Adriatic countries and additionally (2) between three consecutive summer seasons (2020–2022). Because governments acted inconsistently (Škare et al., 2021; Vardavas et al., 2021), we assume similar practices in hotels, which was also reflected on their website communication. Hence, the Adriatic region faced several waves of the pandemic, but nevertheless managed to ensure the crises operation of hotels and communication in the summer seasons.

## Communicating health-related safety in tourism and hotel industry

Corporate communication within crisis management plays an important role in relations with other stakeholders (Coombs, 2019; Dhanesh and Sriramesh, 2018; Su et al., 2019). Companies faced with different situations and reactions to the crises (Ham and Kim, 2019) seek to preserve or fortify credibility and confidence in the eyes of others (Çakar, 2018; Jiang and Wen, 2020) to minimise damage to the image or reputation (Dhanesh and Sriramesh, 2018; Tkalac Verčič et al., 2019). In terms of crisis communication, companies thus follow Coombs’ (2019) situational crisis communication theory (SCCT), which includes links between crisis response strategy and responsibility.

Crisis communication research frequently uses SCCT as the theoretical framework (Cheng, 2020), and because this also applies to the hotel-related research (see Atasoy et al., 2022; Kwok et al., 2021; Wong et al., 2021; Zizka et al., 2021) this study contributes to this trend. SCC theory describes the variables, assumptions and relationships within strategies in times of crisis when organisations should respond responsibly and not routinely to a crisis, which may ultimately be related to the organisation's reputation. The heterogeneity of crises and the organisations involved require different responses with the aim of maintaining reputation with stakeholders; their trust in the messages conveyed is crucial (Coombs, 2004, 2017, 2019; Coombs and Holladay, 2002; Tkalac Verčič et al., 2019). Accordingly, SCCT assumes three crisis clusters from which responsibility for the crisis emerges (victim, accidental and preventable), which dictates managers to choose the appropriate strategy for the right cluster and inform/communicate stakeholders accordingly (Coombs and Holladay, 2002). Moreover, Kwok et al. (2021: 4) draw on previous research and summarise the following SCCT response strategies: denial posture (attacking, denying, scapegoating the accuser), diminishment posture (excusing, justifying), rebuilding posture (making amends, apologising) and bolstering posture (reminding, ingratiating, victimising). This is an organisation-centred and mechanical or organismic perspective (Schultz and Raupp, 2010).

The present global health crisis has been led to a global need for rapid changing/adjustment of the tourism industry. One such change/adjustment is new/different information on COVID-19 (Lee, 2020), including up-to-date information on destinations (Mussini, 2020; Petruzzi and Marques, 2022) to help tourists plan and take trips/holidays. Quintal et al. (2010) found that ‘uncertainty avoidance was positively related to the extent of information search’. The significant/crucial impact of health-related risks in the choice of destination and/or tourism provider cannot be ignored (Bratić et al., 2021; Shin and Kang, 2020); people of different nations do not experience the travel-related risks in the same way (Bratić et al., 2021; Neuburger and Egger, 2021; Torres et al., 2021). Consequently, service providers must make a major effort to reduce unfamiliarity (ignorance) and feelings of anxiety among potential tourists (Bratić et al., 2021); mistrust (Hsieh et al., 2021) and perceived risk play an important role in tourists’ behaviour (Moutinho, 1987; Torres et al., 2021), including travel intentions (Chiu et al., 2019; Karl et al., 2020) and rational decision making (Kim et al., 2023; Torres et al., 2021). The reasonable consequence is that potential tourists who perceive risk may need further information to adjust or cancel their travel-related plans (Bratić et al., 2021; Hassan and Soliman, 2021; Petruzzi and Marques, 2022).

The COVID-19 pandemic is the ‘victim crisis’ for which the hotel industry is not responsible. Hotels can therefore use the bolstering communication strategy to build a positive relationship with their stakeholders (Atasoy et al., 2022; Kwok et al., 2021; Zizka et al., 2021), especially (potential) guests (Hsieh et al., 2021). Zizka et al. (2021: 443) additionally explain that (hotel) organisations ‘should implement an ethical base response consisting of instructing information (i.e. explaining the crisis to the stakeholders) and adjusting information (i.e. helping stakeholders cope with the crisis)’.

The long-lasting and dynamic (phases/waves) of the COVID-19 pandemic with the blurred boundaries between the phases (Hao et al., 2020) makes life and business difficult. Within this context, governments reacted differently with short- and long-lasting measures to manage shocks/crises (Alemanno, 2020; Campos-Rudinsky and Undurraga, 2021; Škare et al., 2021; Vardavas et al., 2021). However, greater certainty in understanding the pandemic resulted in refined measures in individual phases/waves of crisis (Campos-Rudinsky and Undurraga, 2021; Capon et al., 2021). Accordingly, authorities commenced easing travel restrictions, although the border crossing in Europe was not completely free (COVID-19 passports) (Dye and Mills, 2021; Osama et al., 2021); the border crossing was liberalised shortly before the summer of 2022 (*European Union*, n.d.). Furthermore, 2020–2022 pandemic period in the ‘new normal’ helped providers survive and aware tourists could enjoy the summer holidays despite many restrictions. Among other relevant topics, the research on corporate crisis communication (or SCCT) in terms of changing circumstances and government measures is thus justified. With special emphasis on the hotels and their website crisis tourism-centred communication, it will be further discussed in the next section.

### Anti-COVID-19 measures on hotel websites as crisis communication

The sensitivity of the hotel industry is marked by its inclusion into the chain of infection (Cherry, 2004; Yip et al., 2009), which means that preventive measures by hoteliers are of paramount importance. Hao et al. (2020) listed the following hygiene and sanitary measures taken in the COVID-19 pandemic: ‘conducting complete disinfection, controlling food hygiene, distributing masks, offering online medical consultation, detecting the health of customers and employees, and shutting down laundry rooms, gyms, and other public areas and facilities’. Measures should be reflected in the adjusted communication with necessary explanations to restore guests’ confidence (del Valle, 2020); open (Henderson and Ng, 2004; Liu and Pennington-Gray, 2015) and timely communication about safety/security enhance hotel guests’ confidence (Chan and Lam, 2013; Kim et al., 2022) and makes their decisions on accommodation during the travel/holiday easier (Jonas et al., 2011; Liu and Pennington-Gray, 2015). Hotels provide health-related online information in different ways (Kim et al., 2022), including the news media (Le and Phi, 2021; Liu and Pennington-Gray, 2015), social media (Kwok et al., 2021; Liu, Pennington-Gray and Klemmer, 2015; Park et al., 2018) and/or directly on the hotel company websites (Wong et al., 2021; Zizka et al., 2021). As Mohammed et al. (2016) summarise, the latter are effective and direct communication and distribution channels, a source of information as well as booking platforms in contemporary digital age.^1^ As (potential) guests also read the information available there and (then) make a booking, scholars have recommended that hotels post crisis-related messages on their websites (Austin et al., 2012; Zizka et al., 2021). This is completely compatible with the above-mentioned bolstering communication strategy (Atasoy et al., 2022; Kwok et al., 2021; Zizka et al., 2021), which aims to build a positive relationship with (potential) hotel guests (Hsieh et al., 2021) in a certain ethical way (Zizka et al., 2021: 443).

Guest also expect a safe/secure environment during their accommodation when they expect effective reactions (Liu, Pennington-Gray, Donohoe, et al., 2015; Liu and Pennington-Gray, 2015; Petruzzi and Marques, 2022). Consequently, this forces hotel companies to be customer-centric, digital, agile and sustainable (del Valle, 2020) in management, service delivery and communication (Kim et al., 2022). Despite the growing body of literature about pandemics and crisis communication (including SCCT), the present literature review found a lack of research on anti-COVID-19 measures communicated through the corporate website. Moreover, previous research focuses on corporations including hotel chains (Atasoy et al., 2022; Kwok et al., 2021; Wong et al., 2021) and high-class hotels (Zizka et al., 2021), while neglecting independent and lower-classified hotels (including those on the coast) that do not have such managed/systematic communication (Mohammed et al., 2016; Serra-Cantallops et al., 2021). This can put them at a disadvantage in a highly competitive international market with aggressive online promotion and PR/communication by the bigger players (international corporations), see Mohammed et al. (2016). The triangle of website communication–independent hotels–the pandemic crisis therefore deserves the attention of researchers. The following section will highlight the case of the Adriatic accommodation industry, which, along with the health crisis, represents the specific geographical context of this research, on which an empirical analysis will be based.

### Adriatic accommodation industry and COVID-19

According to UN WTO (2018), Europe is the leading contributor to the global tourism industry, and the Mediterranean, of which the Adriatic is a part, is the world's leading tourism area. The coastal stretch is characterised by special hotels (Pons, 2009) – beach or coastal hotels – which, like all tourism there, are very seasonal (Laškarin Ažić et al., 2022; Obadić and Pehar, 2016). The strong dependence on the sea and the summer season in general has a ‘negative impact’ on management and operational processes, including (website) communication with stakeholders.

Taking into account ‘nights spent by domestic and international guests at tourist accommodation establishments per inhabitant’ for 2019 (*Eurostat*, 2021*a*), the three Adriatic countries (Croatia, Slovenia, and Italy) are distinguished by high levels of tourism; they are among the top ten in EU. Croatia (−73.0%), Italy (−56.9%), and Slovenia (−49.4%) were among highly affected countries in the pandemic year 2020 (*Eurostat*, 2021*b*). This also shows that the affected Adriatic accommodation sector (including hotels) can be a relevant subject of academic research.

The perspective of specific beach hotels in the context of the long-lasting and dynamic health crisis and in the context of the within Coombs’ (2017) bolstering communication strategy (including the ethical base response (Zizka et al., 2021)) has not yet been detected in the previous studies. This also relates to hotel company website communication, previously explained by Wong et al. (2021) and Zizka et al. (2021). In addition, scholars suggest more research on crisis communication and COVID-19 (Wong et al., 2021), on different stages of the pandemic and different types of hotels (Atasoy et al., 2022; Hsieh et al., 2021; Zizka et al., 2021), which leads to the following research question:

RQ1: Are there differences in hotel's website crisis communication content between the three pandemic summer seasons?

Due to the already mentioned inconsistent governmental measures to combat the pandemic (Alemanno, 2020; Campos-Rudinsky and Undurraga, 2021; Škare et al., 2021; Vardavas et al., 2021), we assume that the beach hotels in the different Adriatic countries also responded differently/inconsistently, which is also reflected in their crisis communication on the website. In the absence of such international comparative studies (Zizka et al., 2021), Kuščer et al. (2021) recommend comparison between destinations or countries (Hsieh et al., 2021). Based on these considerations, we pose the second research question:

RQ2: Is it the communication of regulations and guidelines via websites associated with the country where the hotel is located? If yes, how strong is this association?

## Material and method

**Figure 1.**
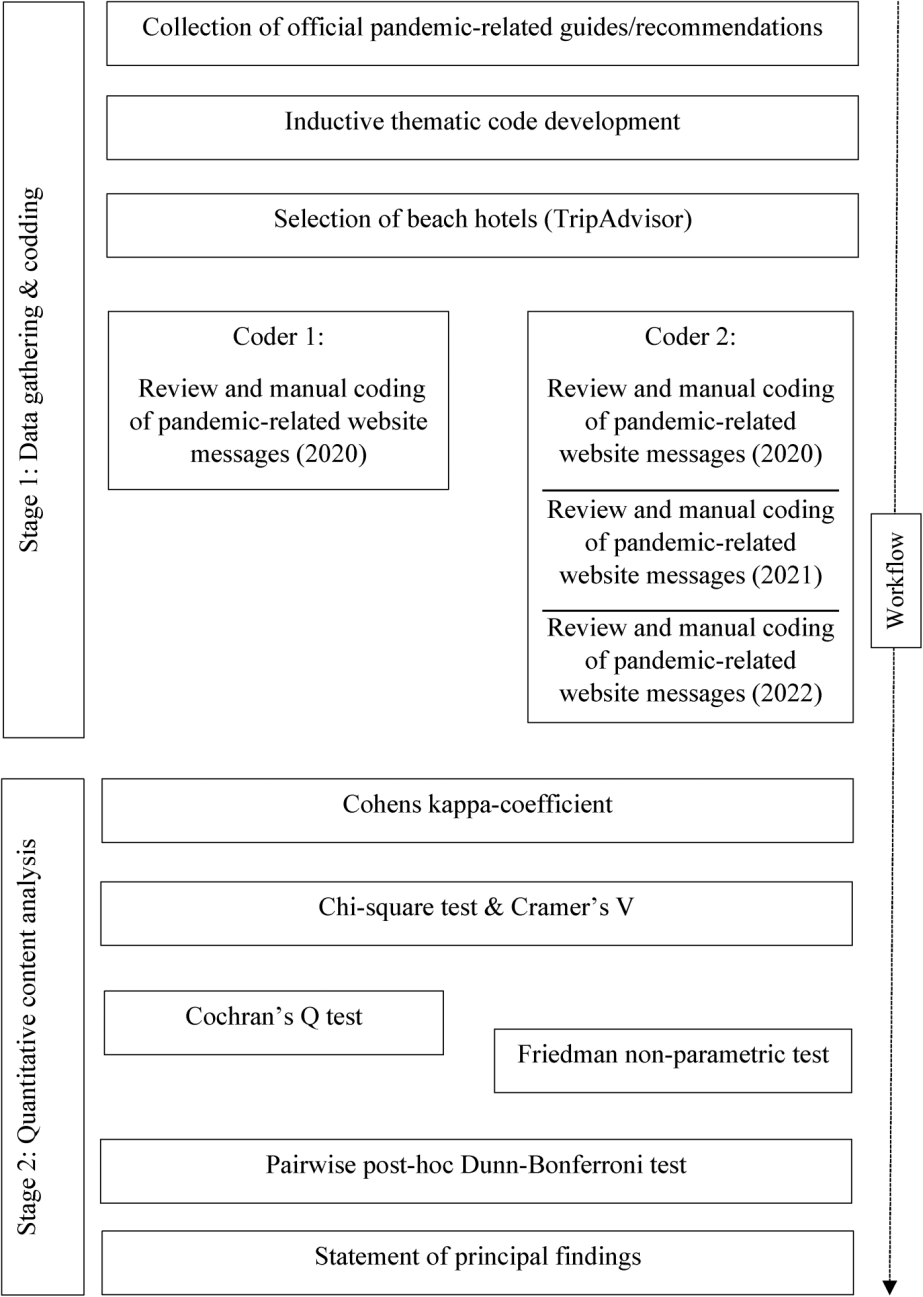
An overview of the research process.

Data gathering and analysis was a complex two-stage process consisting of several substeps (Figure 1). Accordingly, a qualitative inductive thematic code development to identify key themes was applied first. This process relied on the purposive list of official pandemic-related guides/recommendations presented in Table 1; subsequently, compatibility with the ‘Association of Hotels, Restaurants, Pubs and Cafes and similar establishments in Europe’ (*HOTREC*, 202*0*) guidelines and the claims of Hao et al. (2020) was confirmed. To identify the common key themes, the broader codes were initially determined and then synthesised to delineate fewer content categories (as Weber (1990) suggests); the final selection of nine thematic codes is shown in Table 2. Their implementation is explained below.

**Table 1.** Documents for code development.

Organization/Publisher	Document	Published in
World Health Organization	Operational considerations for COVID-19 management in the accommodation sector – Interim guidance	April 2020
European Commission	EU Guidance for the progressive resumption of tourism services and for health protocols in hospitality establishments – COVID-19	May 2020
The World Travel & Tourism Council (WTTC)	Leading global protocols for the new normal – hospitality	May 2020
National agency for tourism, Italy	Guidance standards for hospitality reopening	May 2020
Croatian institute for public health	Recommendations for hotels during COVID-19 epidemic^a^	May 2020
Slovene National institute for public health	Hygiene recommendations for SARS-COV-2 infection prevention in tourism and hospitality industry^a^	September 2020
Insitute for public health of Montenegro	Interim recommendations for action and implementation of protection measures in hotels (hotel industry) and tourism sector^a^	Jun 2020

^a^ Translated title.

**Table 2.** Thematic codes and inter-coder reliability.

Thematic codes	ID	Description	κ
Reference to government guidelines	C1	Procedures, guidelines and protocols developed and presented by health authorities, cooperation with health authorities	1.000
Infection prevention precautions	C2	Hand disinfection, respiratory etiquettes, social behaviour, cleanliness and disinfection measures, frequent touching points disinfection/cleaning, communal areas cleaning and disinfection, cleaning and disinfection innovation, ventilation and airing, preparedness/prevention plan, signage, reservations and large events cancellation, contacts of responsible person or health authority.	1.000
Hygiene equipment	C3	Infection prevention equipment, personal protective equipment usage	1.000
Staff management	C4	Responsibilities of staff, decrease the presence of staff, training of staff, rotation of staff, casual staff minimization workplace adjustment	1.000
Monitoring & supervision	C5	Monitoring compliance: continuous monitoring, monitor and promote the compliance, list of relevant incidents	1.000
Personal communication	C6	Regular communication with guests: communication with customers, digital/electronic means of communication, informational leaflets, promotional materials upon request	0.727
Guidelines provision to guests	C7	Information and guidelines provision to guests, responsible behaviour by guests, elevators usage measures, managing guest contacts, handling COVID-19 infected guests, staying in same room policy	0.710
Departments prevention guidelines	C8	Each hotel department has its own specific measures	1.000
Electronic hotel operations	C9	Electronic payment, auto Check-in and Check-out	0.710

The search for hotels on the TripAdvisor Official Site was done; TripAdvisor as a source is well explained by Egger et al. (2016). The selection consisted of beach hotels that were best rated by the guests. Hence, hotels identified as part of international corporations/brands with sophisticated websites (also checked in the ‘company description’), which are generally less likely to be found on online booking platforms, were excluded. Top ten rated hotels were taken from each coastal region in Adriatic countries. Characteristics of selected hotels are summarised in Table 3.

**Table 3.** Characteristics of hotels (2020).

Hotel characteristics		*f*
*Location (Country)*	Region	
Slovenia	Obalno-Kraška ^a^	6
Bosnia-Herzegovina (B&H)	Herzegovina-Neretva Canton ^a^	4
Italy	Abruzzo ^a^	9
	Apulia	10
	Emilia – Romagna	10
	Friuli – Venezia Giulia	10
	Marche	10
	Molise ^a^	5
	Veneto ^a^	8
Croatia	Dubrovnik and Neretva	10
	Istra	10
	Primorje – Gorski Kotar	10
	Šibenik – Knin	10
	Split and Dalmacija	10
	Zadar ^a^	8
Montenegro	Bar ^a^	7
	Budva	10
	Herceg Novi ^a^	5
	Kotor	10
	Tivat ^a^	8
	Ulcinj ^a^	6
Albania	Durres	10
	Lezhe ^a^	5
	Shkoder ^a^	3
	Tirana	10
	Vlore ^a^	3
*Hotel category*	1 star	6
	2 stars	7
	3 stars	55
	4 stars	107
	5 stars	32
*No. of rooms*	x̄/Med	82.27/48
*Additional services*	SPA	100
	Conference facilities	68
	Sport facilities	77
	Gambling and/or other entertainment facilities	40
	Other	45

^a^ Less than 10 hotels met the abovementioned criteria.

Similar to the research Schultz et al. (2012) conducted in other crises, information about the measures taken to protect health during the COVID-19 pandemic have been sought on hotel websites (the unit of analysis). In fact, they were manually coded using dichotomous thematic codes developed within the first stage (see Table 2). The coder made Yes-No decisions when perceiving codes in website announcements. This coding and additional empirical analyses were implemented within a quantitative content analysis (Camprubí and Coromina, 2016; Riffe et al., 2005), which is understood as an umbrella term (Figure 1).

The first observation was conducted by two coders separately in the first half of July 2020. The inter-rater agreement of the coders was examined at the end of this stage by the Cohens kappa-coefficient (κ). It was found that 1.000 dominates (Table 2). High κ indicates statistically significant substantial or perfect degrees of agreement between coders (Landis and Koch, 1977). Consequently, in the following seasons (the first half of July 2021 and 2022), the coding was done by only one coder. A similar practice of the two coders was found in Liu & Pennington-Gray (2015). While content analysis are primarily used in communication studies (Cheng, 2020), those based on repeated measures are still very rare.

The *χ*^2^ and φ_c_ (Cramer's V) were employed to measure the association between country and website announcements. A Fisher exact test for an r × c contingency table was used (Mehta and Patel, 1983) (RQ2). The non-parametric Cochran's Q-test (Cochran, 1954) was employed to measure the quantitative variability of the website announcements between three consecutive summer seasons. As hotels are understandably not evenly distributed across countries with coastlines of different lengths, the data were previously weighted on this basis – the Cochran's (1954) recommendation. Hence, a non-parametric Friedman test was then used to identify differences in communication content between the three summer seasons. In both cases, the Dunn-Bonferroni post-hoc test was additionally used (RQ1). A statistical significance level of 0.05 was taken into account.

## Results and discussion

After the degree of agreement was confirmed by κ, a validity was checked; the guidelines of Riffe et al. (2005: 169–170) were followed. All available announcements was included in the content analysis, which indicates high external validity/generalisability. External validity can also be evaluated through the scientific relevance of the topic, which can also be confirmed in connection with the literature review in the first part of the paper. In addition, the construct validity derives predominantly from the mixing of the pandemic/crisis, crisis communication and hotel industry theory, for which the findings of recognizable scholars were cited.

**Figure 2.**
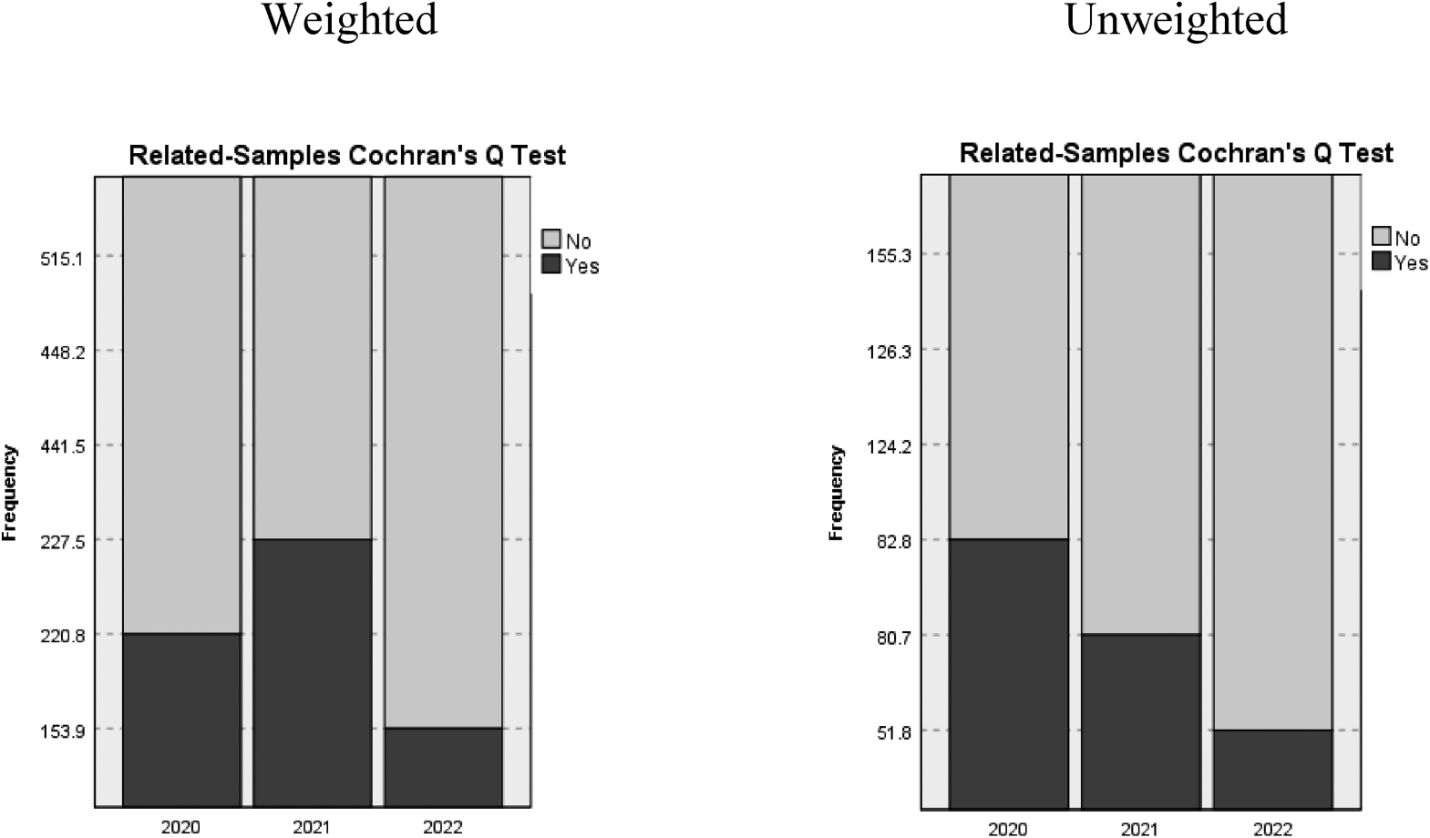
Comparison of the dependent samples between the summer seasons.

Exactly 83 of 207 heterogeneous beach hotels in 2020 communicated their anti-COVID-19 measures via their corporate websites, while this number further decreased until 2022 (see Figure 2, right histogram and Table 4). Table 4 shows significant differences between hotel communication practices among the Adriatic countries: for example, Croatian beach hotels mostly communicate the measures online/website, while in Montenegro and Albania, this option is largely ignored. Using *χ*^2^ and *φ*_c_, we found that the communication of anti-COVID-19 measures is statistically significantly associated with the countries where the hotels are located, exact *p* = 0.000 (2-sided), see Table 4. Indeed, *φ*_c_ ≥ 0.423 proves relatively strong associations (Rea and Parker, 2005) in each of the summer seasons tested. Table 4 also shows some changes in the practise of crisis communication via the website during the three pandemic summer seasons.

**Table 4.** The strength of association between country and communication practice (2020–2022).

Codes	Det	Country						
		Slovenia	Italy	Croatia	Montenegro	Albania	B&H	∑
Regulations ^a^ (2020)	Yes	4	28	41	9	1	0	83
	No	2	34	17	37	30	4	124
	∑	6	62	58	46	31	4	207
Exact *p*	0.000							
*φ*_c_	0.508							
								
Regulations ^a^ (2021)	Yes	3	24	43	9	2	0	81
	No	3	38	15	37	29	4	126
	∑	6	62	58	46	31	4	207
Exact *p*	0.000							
*φ*_c_	0.512							
Regulations ^a^ (2022)	Yes	0	12	30	9	1	0	52
	No	6	50	28	37	30	4	155
	∑	6	62	58	46	31	4	207
Exact *p*	0.000							
*φ*_c_	0.423							

^a^ Regulations and Guidelines communicated via hotels’ website.

In the following step, the quantitative variability of the communication of anti-COVID-19 measures on websites was tested between three consecutive summer seasons. The Cochran's Q-test of weighted dichotomous data (Figure 2, left histogram) indicates a statistically significant difference between seasons: *χ*^2^ = 57.027, *p* = 0.000 (exact *p* = 0.000). The results of the post-hoc Dunn-Bonferroni test (Table 5) show that only the 2022 season is statistically different from the previous two seasons (*p* = 0.000); as mentioned earlier, 2022 had the fewest announcements.

**Table 5.** Pairwise comparison between summer seasons.

Sample 1-Sample 2	Test statistic	Std. error	Std. test statistic	Sig.	Adj. Sig.
Y2022-Y2020	0.108	0.017	6.448	0.000	0.000
Y2022-Y2021	0.111	0.017	6.628	0.000	0.000
Y2020-Y2021	−0.003	0.017	−0.179	0.858	1.000

**Figure 3.**
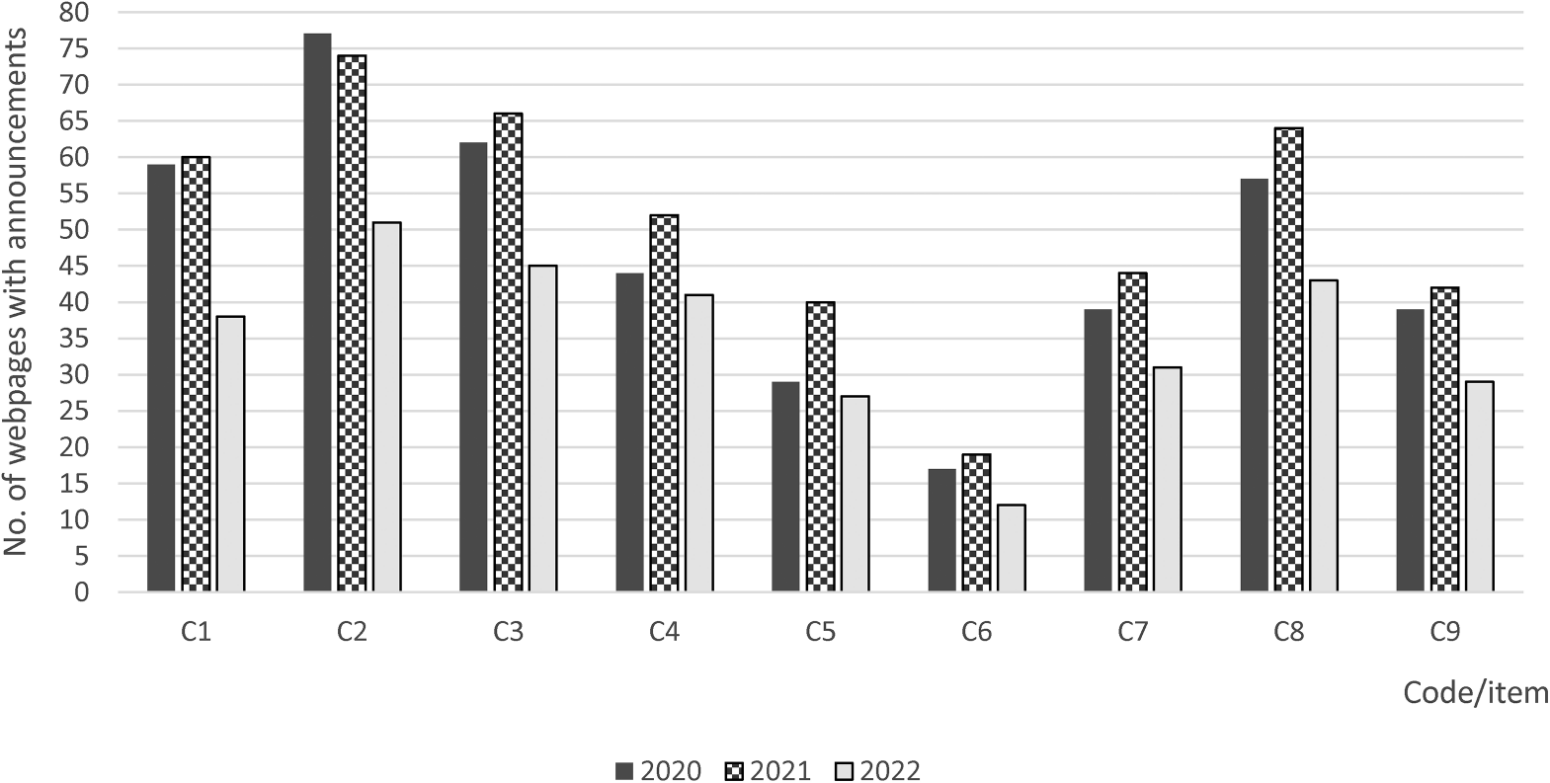
Differences in codes/items between analysed summer seasons.

Hotels that communicated their measures via the website (marked with ‘Yes’) were additionally analysed: the differences in the content of communications/announcements between three consecutive summer seasons have been tested. Figure 3 shows a graphical representation of the totals for each code/item by year/summer season (see also Table 2). It can be seen that ‘Infection prevention precautions’ (C2) is the most common topic of communication, while the content related to ‘Personal communication’ (C6) was by far the least used. Furthermore, there is a significant decrease in all items in the 2022 season. The Friedman test confirms a statistically significant difference between the seasons: *χ*^2^ = 16.222, *p* = 0.000 (exact *p* = 0.000). A post-hoc Dunn-Bonferroni test (Table 6) shows that the 2022 season is statistically different from the previous seasons, although the difference between 2020 and 2022 is marginal (adj. *p* = 0.055).

**Table 6.** Pairwise comparison between summer seasons in communication content.

Sample 1-Sample 2	Test statistic	Std. error	Std. test statistic	Sig.	Adj. Sig.
Y2022-Y2020	1.111	0.471	2.357	0.018	0.055
Y2022-Y2021	1.889	0.471	4.007	0.000	0.000
Y2020-Y2021	−0.778	0.471	−1.65	0.099	0.297

The above results show that only about 40% of Adriatic beach hotels in 2020 communicated their anti-COVID-19 measures via their website (Table 4); by 2022 this proportion decreases significantly in all countries, even though the health crisis was not yet over (*United Nations*, 202*2*). Thus, they neglect/ignore or underestimate the importance of this kind of communication (Ettinger et al., 2018; Koronios et al., 2021; Petelin and Križaj, 2020) during the crisis. This ‘ignore strategy’ of crisis communication represents a departure from SCCT as defined by Coombs (2004, 2017, 2019; Coombs and Holladay, 2002). Similar findings were reported by Zizka et al. (2021) in the case of Swiss 4 and 5-star independent hotels, which clearly indicates the specific crisis communication of hotels during the COVID-19 crisis. However, this is not a peculiarity of the hotel industry, as this simple solution has already been observed during the Arab Spring uprisings (Avraham, 2015); some non-tourism examples can be found in Liu (2010). ‘Ignore the public’ approach was in health risk issues otherwise prevalent in the USA until the late 1960s (Covello, 2021: 73). Moreover, Coombs and Holladay (2015) highlighted this scenario by discussing corporate social responsibility and a crisis risk. Nevertheless, not all of these cases had such a global dimension as the pandemic COVID-19. Significant differences between countries have been identified within this study: communication of anti-COVID-19 measures is statistically significantly associated with the countries in which the hotels are located (RQ2). Hotels in less-developed countries (*World Economic Situation Prospects*, 2020), such as Montenegro and Albania, almost completely neglect these modern communication channels; in economically developed Italy, at least in 2020, use was significantly better but less frequent than in the Croatian beach hotels. An otherwise small selection of hotels from the short coastlines of Slovenia and less developed B&H indicate different practices, especially in 2020 (Table 4). Thus, in the absence of research on crisis communication via hotel websites, this study shows that the transformation of hotel businesses’ (Hao et al., 2020; Le and Phi, 2021) into customer-centric, digital, agile, and sustainable (del Valle, 2020) were only partially considered in the case of the Adriatic beach hotels. Traditional considerable dependence on tourism of the Adriatic countries (*Eurostat*, 2021*a*), the negative consequences of the pandemic in the accommodation sector in the region (*Eurostat*, 2021*b*), measures of governments (Alemanno, 2020; Škare et al., 2021) and the negative consequences of the pandemic for the domicile population in the region (*Worldometers*, n.d.) evidently did not encourage hoteliers to change/adjust models in the field of website crisis communication.

The next issue is the content of hotels’ website crisis communication and its adaptation to changing conditions. As described above, communication via websites in the Adriatic was not a common practice. Advances in science and related changing formal measures (Campos-Rudinsky and Undurraga, 2021; Capon et al., 2021; Osama et al., 2021) are reflected to a limited extent in communication content available on corporate websites. The number of different announcements differs significantly (RQ1) only between the 2021 and 2022 seasons, when formal measures at national levels were instigated (*European Union*, n.d.; *United Nations*, 202*2*). This finding is inconsistent with those of Lee (2020) and Mussini (2020) on up-to-date pandemic-related information, which helps tourists plan and take trips/holidays. Many Adriatic hotels thus do not make enough effort to reduce unfamiliarity/ignorance and feelings of anxiety among potential tourists (Bratić et al., 2021; Hassan and Soliman, 2021; Torres et al., 2021). Furthermore, this negative three-season trend clearly shows that even many hotels that responded with bolstering communication strategies and a grassroots ethical response on hotel websites in the first waves of the pandemic (see also Zizka et al., 2021), switched to the ‘ignore strategy’ of crisis communication in the 2022 season, despite the ongoing health crisis. This suggests a significant/dominant influence of formal government measures on their crisis communication strategies.

## Conclusions

Anti-COVID-19 measures have serious socio-economic consequences for the tourism industry, including the hotel industry. Under these circumstances, researchers, managers, marketers, and experts/consultants need to analyse impacts and responses to minimise crises’ negative consequences. Their findings are important for the theoretical development of the hotel industry's crisis management and communication literature and present practical perspectives to help reduce potential guests’ insecurities and make their decisions during (health) crises easier (Jonas et al., 2011; Liu and Pennington-Gray, 2015; Torres et al., 2021). As previously stated, knowledge of the possibilities offered by corporate websites (Ettinger et al., 2018; Quintal et al., 2010), crisis-communication (Wong et al., 2021) at different stages of the pandemic and in different types of hotels (Atasoy et al., 2022; Hsieh et al., 2021; Zizka et al., 2021), and cases from non-English speaking environments (Le and Phi, 2021; Zizka et al., 2021) are lacking. The present research fills this gap with the case of beach hotels from the internationally recognizable tourism area.

Accordingly, the present paper makes significant theoretical contributions to the body of knowledge in situational crisis communication and COVID-19 by exemplifying the website crisis communication of (beach) hotels. To date, very few quantitative studies have been conducted on tourist-centred crises and website communications in the hotel industry, which is reflected in many knowledge gaps. The results show that communication of anti-COVID-19 measures is statistically significantly associated with the country in which hotels are located. Using the example of specific beach hotels in the Adriatic countries, we found that not only governments act differently, but that this was also true for beach hotels and their crisis communication on the website (RQ2). Hence, we also identified the content of hotels’ website communication as well as seasonally different website communication practises, with the predominant ‘ignore strategy’ being particularly evident in the 2022 summer season, even though the pandemic was not yet over (RQ1). This ‘ignore strategy’ of crisis communication represents a departure from SCCT as defined by Coombs (2004, 2017, 2019; Coombs and Holladay, 2002). It has also been found in some previous research (e.g. Avraham, 2015; Coombs and Holladay, 2015; Liu, 2010; Zizka et al., 2021), which clearly points to a legitimate need to revise SCC theory; this study thus offer a useful contribution to this challenging process. This study also highlights the importance of examining crisis communication over a longer period of time with repeated measures, which have been overlooked so far. This research also confirms how important are concrete cases, including those that are at the top of world tourism (e.g. the Adriatic within the Mediterranean), in the study of crisis communication in specific contexts and periods.

At this point, we can connect to some practical implications of this research. Firstly, managers (and marketers) should take the necessary steps to invest in ICT and website crisis communication and establish a supportive environment to transfer good practices from Croatian hotels (2020 and 2021 seasons), for example, which were the most propulsive in this field in the region. The digitization of communication that has only been accelerated by the COVID-19 pandemic, must also include training of managers and staff. National/regional/local professional associations representing hotels and/or other relevant development organisations should promote and support this in order to make it easier for (smaller) independent hotels to start such projects; this is particularly relevant for economically weaker countries/destinations. With the adequate hardware, software and knowledge, they can realise potentials of websites and other online channels for communicating with (potential) guests and other stakeholders before, during and after crises, especially as the effects of the ‘ignore strategy’ used in crisis communication have not yet been explained. Secondly, website crisis communication should follow measures of governments and public health institutions until the crisis is over,^2^ which will not be a question for competent managers and staff. This way, unfamiliarity and feelings of anxiety among potential tourists can be reduced, and decision-making facilitated.

By focusing on the Adriatic basin, the limited scope of this research is recognizable. Due to its diverse structure (Table 3), the hotel industry studied in a coastal area involved in international flows makes it a good case study, the results of which can be valuable for other regions/destinations (particularly seasonal ones). In particular, for independent hotel owners and/or managers, marketers and hotel website developers, as well as public health experts and political decision-makers, the findings can be used to develop effective online crisis communication in times of health crises. However, the different and inconsistent responses of governments to health crises (Hsieh et al., 2021; Škare et al., 2021), the impact of national cultures on crisis communication (Dhanesh and Sriramesh, 2018) and the significant differences in hotel industries among countries/destinations (e.g. in different experiences/practices, sector structure, locations, hotels’ penetration rates (Carvell et al., 2016)), limit the easy transferability of results to the wider global scale. Future research can further clarify this issue by building on the cultural dimensions of Hofstede et al. (2010).

An additional limitation is that only beach hotels present on TripAdvisor that benefit from the summer season have been included. Coastal destinations also offer other accommodations, and other online travel platforms also exist. Future research may take this into account as well as new post-pandemic business models/approaches in the hotel industry, see Chang et al. (2022). Nevertheless, the developed measuring instrument and approach are useful for any hotel type, regardless of platform, communication channel, location, and season. In addition, empirical analysis with binary items provides information on how hotels communicate through websites and ignore the perspectives of guests and other stakeholders that are otherwise not overlooked by SCC theory; the need for its revision has already been mentioned. Finally, similar research in a different context (e.g. different seasons, destinations, crises and post-crisis period) where other accommodation establishments and service providers should be conducted to compare with the current findings.

## Notes

1. Hotel company websites were also explored before the pandemic COVID-19, see Petelin and Križaj (2020), Ettinger et al. (2018) and Mohammed et al. (2016). This shows that this channel/topic is not marginalised by international researchers. In times of pandemic, this issue/topic has become even more important.

2. In accordance with national laws and international conventions, governments and the World Health Organisation also decide on this.
